# Protective Efficacy of a Hemagglutinin-Based mRNA Vaccine Against H5N1 Influenza Virus Challenge in Lactating Dairy Cows

**DOI:** 10.34133/research.1104

**Published:** 2026-01-26

**Authors:** Huihui Kong, Jiaxin Yang, Jianzhong Shi, Pengfei Cui, Xianying Zeng, Wenyu Liu, Xijun He, Xianfeng Zhang, Lei Chen, Yichao Zhuang, Yan Wang, Jinming Ma, Jiaqi Li, Yaping Zhang, Congcong Wang, Chen He, Jiongjie Li, Jinyu Yang, Jinxiong Liu, Pucheng Chen, Yuntao Guan, Zhigao Bu, Yongping Jiang, Hualan Chen

**Affiliations:** ^1^ State Key Laboratory of Animal Disease Control and Prevention, Harbin Veterinary Research Institute, Chinese Academy of Agricultural Sciences, Harbin 150069, People’s Republic of China.; ^2^ National High Containment Laboratory for Animal Disease Control and Prevention, Harbin 150069, People’s Republic of China.; ^3^ Harbin Weike Biotechnology Co. Ltd., Harbin 150069, People’s Republic of China.

## Abstract

Highly pathogenic avian influenza H5N1 virus has spread to over 1,080 dairy farms across 18 states in the United States, resulting in 41 human infections and posing serious risks to both animal and public health. To address these risks, a hemagglutinin-based mRNA–lipid nanoparticle vaccine was developed, and its safety, immunogenicity, and protective efficacy in high-yielding lactating dairy cows were evaluated. The vaccine was well tolerated, had no adverse effects on health or milk production, and induced strong antibody responses. Two weeks after the second immunization, all the immunized cattle were fully protected against a high-dose H5N1 virus challenge. Notably, two-thirds of the cattle were still completely protected even at the 19th week after the first vaccination, when their serum antibody levels were very low. These data demonstrate that the mRNA vaccine confers robust, lasting protection against H5N1 virus in lactating dairy cows, providing a foundation for clinical trials.

## Introduction

The highly pathogenic avian influenza (HPAI) H5N1 virus was first detected in lactating dairy cows in the United States on 2024 March 25 [1]*.* In these outbreaks, the most harmful characteristic of H5N1 influenza outbreaks in dairy cattle is the viral invasion of and damage to the mammary glands, which has primarily been attributed to “mouth-to-teat” transmission during self-sucking or cross-sucking behaviors among cows [2,3]. Since its initial emergence, the virus has spread throughout the United States rapidly, leading to outbreaks on over 1,080 dairy farms across 18 states and resulting in 41 confirmed human infections as of 2025 November 20 [1]. Accordingly, this virus poses a significant risk to the dairy industry and to public health [4,5]. Phylogenetic analyses revealed that the dairy cow H5N1 viruses circulating in cattle belong to clade 2.3.4.4b, a clade that poses a major global concern due to its severe impact on poultry, wildlife, and potential risks to human health [6–9]. Two distinct genotypes, B3.13 and D1.1, have been isolated from outbreaks in infected cattle [10]. Both originated from reassortment events between low pathogenic avian influenza viruses carried by migratory wild birds in the Americas and Eurasian avian lineages introduced into North America. Specifically, genotype B3.13 acquired the hemagglutinin (HA), neuraminidase (NA), polymerase acidic, and matrix (M) gene segments from the Eurasian A1 (ea1) lineage, while genotype D1.1 contains the HA, polymerase basic 1 (PB1), M, and nonstructural segments derived from the ea3 lineage [10]. The emergence and spread of H5N1 virus in dairy cows represent a host jump that is unprecedented. Notably, several harmful mutations (e.g., PB2-627K and PB2-701N) that increase the pathogenicity of influenza viruses in humans have been detected in H5N1 viruses isolated from dairy cattle and dairy workers [11,12], which underscores the urgent need to enhance virological surveillance, strengthen farm biosecurity, and accelerate the generation of highly protective vaccines to mitigate the threat that H5N1 poses to the dairy industry and public health [1].

Vaccination is a well-established and effective strategy to control influenza viruses in livestock and to mitigate the risk of viruses with pandemic potential [13–17]. Since 2004, several countries, including China, Mexico, Vietnam, Russia, Bangladesh, Egypt, and Indonesia, have adopted vaccine immunization strategies in poultry to control HPAI H5 virus [13]. These sustained efforts have played a crucial role in reducing HPAI outbreaks and limiting zoonotic spillover events [14]. One prominent example is China’s use of a bivalent inactivated vaccine for chickens that simultaneously targets H5 and H7 HPAI viruses. This strategy effectively curbed H7N9 outbreaks in poultry and prevented subsequent human infections, demonstrating the dual benefits of animal vaccination for public and animal health [18]. In response to the growing threat posed by persistent H5N1 outbreaks, many European and North American countries are now considering or initiating poultry vaccination programs. Germany, Italy, and the Netherlands have already completed vaccination trials [19]. France has taken a leading role by implementing a mandatory preventive HPAI vaccination program for commercial duck farms [20]. In addition, the US Department of Agriculture has conditionally approved an H5N2 inactivated vaccine developed by Zoetis to protect poultry against the circulating H5N1 strain [21]. Moreover, Finland has become the first country in the world to initiate bird flu vaccinations in humans, marking a milestone in pandemic preparedness [22]. Given the success of poultry vaccination and the rising risk of H5N1 in cattle, vaccinating lactating dairy cows could be a practical strategy to prevent virus spread and reduce zoonotic risk.

Multiple vaccine platforms are being explored, such as inactivated, live-attenuated, DNA, and mRNA-based vaccines [23–32]. Shi et al. [2] previously reported that both inactivated vaccine and HA-gene-based DNA vaccines could provide sterilizing immunity in lactating dairy cows against high-dose H5N1 virus challenge. Because of COVID-19, mRNA vaccines have emerged as a particularly attractive option. Several studies have shown that mRNA vaccines are highly effective against emerging H5N1 strains in various animal models [23,25,28,30–32]. Chiba et al. [23] showed that an mRNA vaccine provided complete protection in a mouse model challenged with the newly emerged dairy cow H5N1 strain*.* Hatta et al. [28] reported that H5-specific mRNA vaccination protected animals from fatal infection, although low-level viral shedding in nasal secretions was still observed. Furey et al. [25] tested a vaccine based on nucleoside-modified mRNA in ferrets and observed that the vaccine was protective against challenge with clade 2.3.4.4b H5N1, although some viral shedding in nasal washes persisted. Souza et al. [31] demonstrated that mRNA vaccine induced antibody responses in calves. Despite these encouraging findings in small animal and calf models, no evaluation of mRNA vaccine has yet been conducted in lactating dairy cows, the primary target population for bovine H5N1 vaccine development.

In this study, we developed an mRNA–lipid nanoparticle (LNP) vaccine that encodes the HA protein derived from a clade 2.3.4.4b H5 virus, which is antigenically identical to the strain actively circulating within cattle herds across the United States [2]. Comprehensive evaluation of this vaccine in lactating dairy cows demonstrated its safety, induction of robust and long-lasting immunity, and ability to provide sound protection against high-dose H5N1 virus challenge. Our findings support the promising role of mRNA vaccines in controlling H5N1 infection in lactating dairy cows and mitigating the risk of potential zoonotic spillover.

## Results

### Safety and immunogenicity of an H5 mRNA–LNP vaccine

To construct an mRNA–LNP vaccine suitable for protecting dairy cows against H5N1 infection, we selected the full-length HA gene from an HPAI H5N8 virus (A/whooper swan/Shanxi/4-1/2020), which serves as the donor of surface genes for the vaccine strain H5-Re14. H5-Re14 is currently used in China to protect against clade 2.3.4.4b H5 viruses [33] and is antigenically identical to the dairy cow isolate A/dairy cow/Texas/24-008749-001/2024 (H5N1, DC/24) [2]. We developed a monovalent, cattle-codon-optimized mRNA–LNP vaccine encoding the HA protein of A/whooper swan/Shanxi/4-1/2020 and evaluated its safety and immunogenicity in lactating dairy cows.

**Fig. 1.**
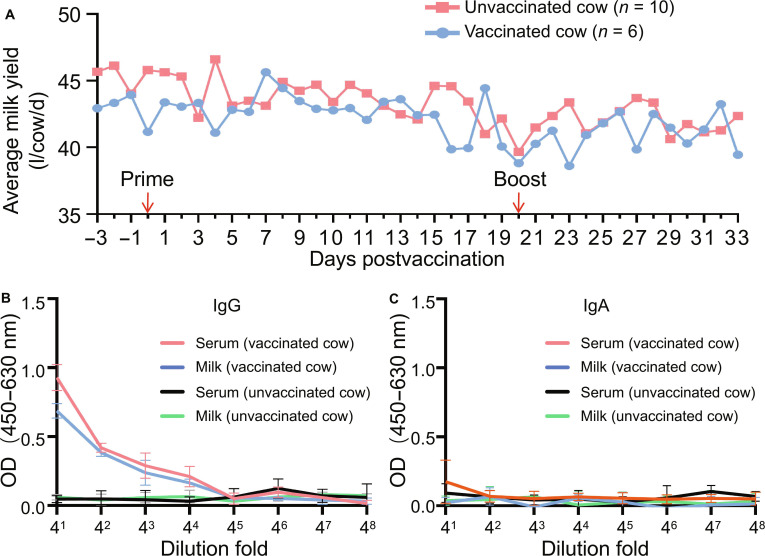
Safety and induced antibody isotypes of the mRNA vaccine in lactating dairy cows. Six cows were immunized intramuscularly with 500 μg of mRNA–LNP twice, 3 weeks apart. (A) Daily milk yield in vaccinated cows and unvaccinated controls, recorded from 3 d before the first immunization to 14 d after the booster. (B and C) Antibody isotypes in serum and milk measured by ELISA:IgG (B) and IgA (C) using bovine IgG- and IgA-specific detection antibodies. OD, optical density.

Six healthy lactating dairy cows were intramuscularly immunized with 500 μg of the mRNA vaccine twice, with a 3-week interval between doses. Milk yield was recorded daily from 3 d before the first immunization until 14 d after the second immunization. As shown in Fig. 1A, the milk production of the vaccinated cattle was comparable to that of the unvaccinated controls throughout the observation period. We also monitored the body temperature and food intake of these cattle but detected no abnormal conditions, indicating that the vaccine is safe for lactating dairy cows.

To characterize the immune responses induced by vaccination, we collected the serum and milk samples weekly or biweekly postvaccination (p.v.), starting 2 weeks prior to vaccination. Following mRNA vaccination, immunoglobulin G (IgG) constituted the major antibody isotype in both serum and milk (Fig. 1B and C). Hemagglutination inhibition (HI) and/or neutralization (NT) titers were measured for each sample. After the first dose, both HI and NT antibodies were induced in serum, with the HI titers ranging from 8 to 128 and the NT titers ranging from 32 to 128 at 3 weeks p.v. (Table 1). A marked increase in HI and NT antibody titers occurred 1 week after the booster dose, followed by a gradual decline in the subsequent weeks (Table 1). The observed dynamic patterns of HI and NT titers were consistent with those previously reported in ferrets by Hatta et al. [28]. The pattern of NT antibody titers in milk was similar to that in serum, with peak titers observed shortly after the booster dose (Table 1). Two weeks after the booster dose, 3 animals underwent viral challenge as outlined below, and the remaining 3 animals were observed for their antibody duration. NT antibodies in milk became undetectable in all 3 cattle at week 15 p.v. (Table 1), whereas at week 19 p.v., the HI and NT antibodies in serum could still be detected in 2 and 3 animals, respectively (Table 1). Collectively, these data demonstrate that the vaccine is safe and elicits robust, long-lasting antibody responses in lactating dairy cows.

**Table 1.** Antibody titers in serum and milk collected from vaccinated cows

Antibody evaluated (sample)	Cow no.^a^	Euthanasia day postchallenge	Antibody titers at different weeks post-prime-vaccination											
			0	2	3^b^	4	5	7	9	11	13	15	17	19
HI (serum)	Cow 1	3	<2	8	8	256	128	–	–	–	–	–	–	–
	Cow 2	6	<2	8	16	512	256	–	–	–	–	–	–	–
	Cow 3	12	<2	32	128	512	256	–	–	–	–	–	–	–
	Cow 4	3	<2	16	32	256	256	128	32	16	16	8	<2	<2
	Cow 5	6	<2	16	32	256	128	64	32	8	8	8	8	8
	Cow 6	12	<2	32	64	512	512	512	128	64	64	32	16	16
NT (serum)	Cow 1	3	<2	32	32	256	256	–	–	–	–	–	–	–
	Cow 2	6	<2	16	32	512	256	–	–	–	–	–	–	–
	Cow 3	12	<2	64	128	1,024	512	–	–	–	–	–	–	–
	Cow 4	3	<2	16	32	256	256	64	64	32	16	16	16	8
	Cow 5	6	<2	16	32	256	512	64	32	16	32	16	16	16
	Cow 6	12	<2	32	32	1,024	512	512	128	64	64	32	16	16
NT (milk)	Cow 1	3	<2	<2	8	32	32	–	–	–	–	–	–	–
	Cow 2	6	<2	<2	<2	64	32	–	–	–	–	–	–	–
	Cow 3	12	<2	<2	4	128	64	–	–	–	–	–	–	–
	Cow 4	3	<2	<2	4	128	32	32	16	4	<2	<2	<2	<2
	Cow 5	6	<2	<2	<2	32	32	16	4	<2	<2	<2	<2	<2
	Cow 6	12	<2	<2	<2	32	32	16	16	8	4	<2	<2	<2

^a^ Cows 1 to 3 underwent viral challenge 2 weeks after receiving the booster dose (corresponding to week 5 after the initial vaccination), while cows 4 to 6 were challenged 16 weeks after the booster (week 19 following the first dose).

^b^ A booster dose of the mRNA vaccine was administered to cows 3 weeks after the initial injection.

Note: “–” indicates not collected.

### Protective efficacy of the mRNA–LNP vaccine

Two weeks after the second immunization (5 weeks p.v.), 3 vaccinated and 3 unvaccinated lactating dairy cows were transferred into the animal biosafety level 3+ (ABSL-3+) facility for the challenge study. The animals were acclimatized for 2 d prior to viral challenge. All cows received a DC/24 virus challenge administered via both the intranasal and intramammary routes, as previously described [2]. Briefly, 2 × 10^6^ EID_50_ (50% egg infectious dose) of virus was administered intranasally (1 ml per nostril), and 3 doses were directly inoculated into separate mammary quarters via the teat: 1 ml (10^2^ EID_50_) into the left rear quarter, 1 ml (10^4^ EID_50_) into the right front quarter, and 1 ml (10^6^ EID_50_) into the right rear quarter. Following challenge, nasal swabs, saliva, and milk samples from each mammary gland were collected daily for viral titration. Clinical signs, including rectal temperature and milk appearance, were monitored throughout the postchallenge observation period. To investigate viral dissemination and tissue-level protection, we humanely euthanized one cow from each group (vaccinated and unvaccinated) on days 3, 6, and 12 postchallenge and subsequently collected tissues from the oral cavity, respiratory tract, and mammary quarters for virus detection and harvested them for virus detection.

**Fig. 2.**
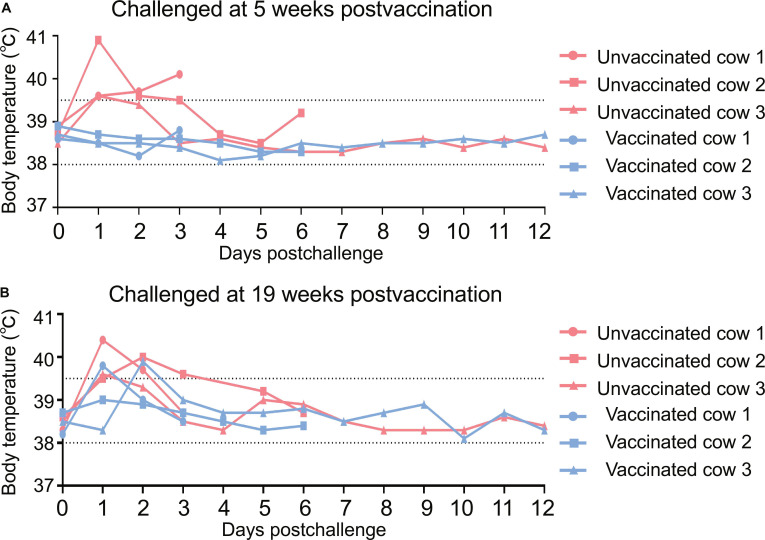
Temperature fluctuations of cows following viral challenge. Vaccinated and unvaccinated dairy cows received a DC/24 virus challenge administered via both the intranasal and intramammary routes. Rectal body temperature was monitored daily postchallenge. (A) Rectal body temperature of cows challenged at 5 weeks p.v. (B) Rectal body temperature of cows challenged at 19 weeks p.v.

**Fig. 3.**
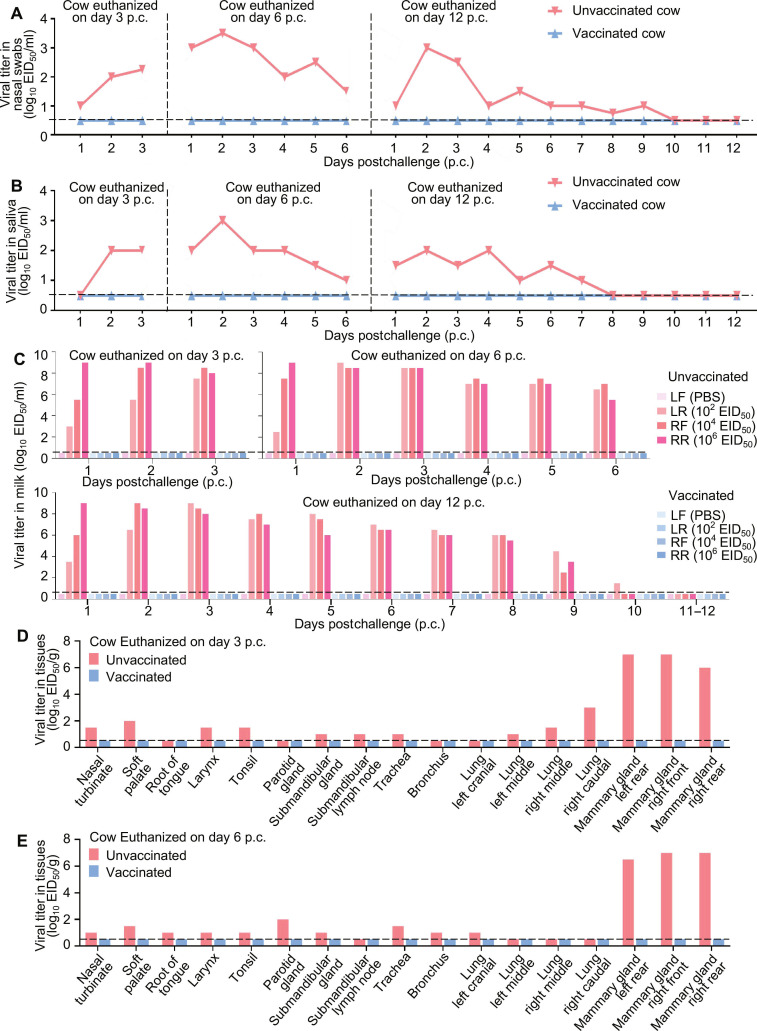
Protective efficacy of the H5 mRNA–LNP vaccine in lactating dairy cows 5 weeks p.v. Vaccinated and unvaccinated dairy cows were challenged with a dairy cow H5N1 virus via intranasal and intramammary routes. A dose of 2 × 10^6^ EID_50_ of virus was administered intranasally (1 ml per nostril), and different doses were administered to individual mammary quarters via the teat: 1 ml of PBS into the left front (LF) quarter, 1 ml (10^2^ EID_50_) into the left rear (LR) quarter, 1 ml (10^4^ EID_50_) into the right front (RL) quarter, and 1 ml (10^6^ EID_50_) into the right rear (RR) quarter. Nasal swabs, saliva, milk, and tissues were harvested at the indicated times postchallenge (p.c.) and titrated in embryonated chicken eggs. All panels show viral titers in (A) nasal swabs, (B) saliva, (C) milk, and tissues (D and E). Each bar denotes an individual cow. The lower detection threshold is shown as a horizontal dashed line.

In the unvaccinated animals, body temperature increase was observed in all 3 cattle after challenge (Fig. 2A), and the milk produced by the virus-inoculated mammary glands turned yellow and colostrum-like as early as day 2 postchallenge; this persisted for up to 6 d before the appearance of the milk returned to milky appearance (Table 2). Nasal swabs from all 3 cows were positive for viral shedding starting on day 1 postchallenge, as well as in the animal culled on day 12 postchallenge, shedding continued for a total of 9 d (Fig. 3A); virus became detectable in the saliva from 2 cows on day 1 postchallenge, and by 2 d postchallenge, all 3 animals were positive. In the cow culled on day 12 postchallenge, oral viral shedding persisted for as long as 7 d (Fig. 3B). High viral titers were detected in milk samples from all inoculated mammary quarters, and in the cow culled on day 12 postchallenge, shedding persisted for up to 10 d (Fig. 3C). In tissue samples of the dairy cows humanely euthanized on days 3 and 6 postchallenge, virus was recovered from the nasal turbinate, tongue root, soft palate, larynx, tonsil, parotid gland, submandibular gland, submandibular lymph node, trachea, bronchus, and 4 lobes of the lungs, in addition to the virus-inoculated mammary glands (Fig. 3D and E), but virus was not detected in any samples from the cow culled on day 12 postchallenge (Fig. S1A). In contrast, the vaccinated cows exhibited no fever, and their milk stayed milky after virus challenge (Fig. 2A and Table 2). None of the nasal swab, saliva, milk, or tissue samples collected from vaccinated cows yielded detectable virus (Fig. 3A to E and Fig. S1B). These data demonstrate that the mRNA–LNP vaccine confers complete protection against H5N1 infection in lactating dairy cows.

**Table 2.** Changes in milk color in dairy cattle after viral challenge^a^

Challenge time	Group	Euthanasia time (day postchallenge)	Milk color in mammary gland (day postchallenge)			
			Left front	Left rear	Right front	Right rear
5 weeks after the first dose of vaccine	Control	3	Milky	Milky	Yellow (3)	Yellow (2 to 3)
		6	Milky	Yellow (4–6)	Yellow (3–5)	Yellow (2–5)
		12	Milky	Yellow (4–6)	Yellow (4–6)	Yellow (2–6)
	Vaccinated	3	Milky	Milky	Milky	Milky
		6	Milky	Milky	Milky	Milky
		12	Milky	Milky	Milky	Milky
19 weeks after the first dose of vaccine	Control	3	Milky	Milky	Milky	Yellow (3)
		6	Milky	Yellow (4–6)	Yellow (3–6)	Yellow (2–6)
		12	Milky	Yellow (3–8)	Yellow (3–8)	Yellow (2–8)
	Vaccinated	3	Milky	Milky	Milky	Milky
		6	Milky	Milky	Milky	Milky
		12	Milky	Milky	Milky	Milky

^a^ Different doses were administered to individual mammary quarters via the teat: 1 ml of PBS into the left front teat, 1 ml (10^2^ EID_50_) into the left rear quarter, 1 ml (10^4^ EID_50_) into the right front quarter, and 1 ml (10^6^ EID_50_) into the right rear quarter.

### Long-lasting protective efficacy of the mRNA–LNP vaccine

At 19 weeks p.v., 2 and 3 animals had detectable HI antibodies and NT antibodies, respectively, in their serum, but none had detectable NT antibodies in milk. To investigate the resistance of vaccinated cattle with declining antibody levels against H5N1 virus infection, we challenged the 3 vaccinated cows and 3 unvaccinated controls using the same viral challenge protocol.

**Fig. 4.**
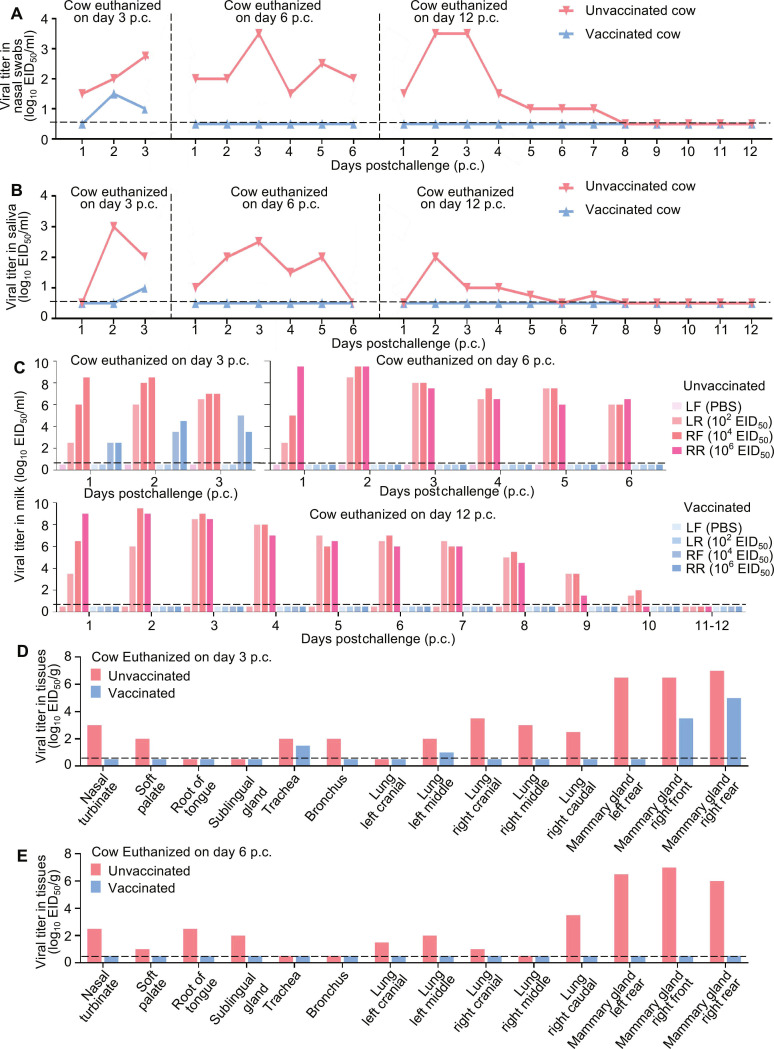
Protective efficacy of the H5 mRNA–LNP vaccine in lactating dairy cows 19 weeks p.v. Vaccinated and unvaccinated dairy cows were challenged with a dairy cow H5N1 virus via intranasal and intramammary routes. A dose of 2 × 10^6^ EID_50_ of virus was administered intranasally (1 ml per nostril), and different doses were administered to individual mammary via the teat: 1 ml of PBS into the left front quarter, 1 ml (10^2^ EID_50_) into the left rear quarter, 1 ml (10^4^ EID_50_) into the right front quarter, and 1 ml (10^6^ EID_50_) into the right rear quarter. Nasal swabs, saliva, milk, and tissues were harvested at the indicated times postchallenge and titrated in embryonated chicken eggs. All panels show levels of virus in (A) nasal swabs, (B) saliva, (C) milk, and tissues (D and E). Each bar denotes an individual cow. The lower detection threshold is shown as a horizontal dashed line.

In the unvaccinated group, fever was observed in all 3 cattle after challenge (Fig. 2B), and the milk produced by the virus-inoculated mammary glands turned abnormal starting on day 2 postchallenge (Table 2). Virus shedding started on day 2 postchallenge in nasal swabs and saliva of all 3 cows, and viral shedding in both nasal swabs and saliva persisted for as long as 7 d in the animal culled on day 12 postchallenge (Fig. 4A and B); viral presence was confirmed in milk samples from every virus-inoculated mammary gland in the 3 animals (Fig. 4C). In cows culled on days 3 and 6 postchallenge, viral replication was observed in multiple tissues, namely, the nasal turbinate, tongue root, soft palate, sublingual gland, bronchus, trachea, lungs, and virus-inoculated mammary glands, but was not detected in any tissues of the animal culled on day 12 postchallenge (Fig. 4D and E and Fig. S2A).

In the vaccinated group, 2 animals developed fever on days 1 and 2 postchallenge, respectively (Fig. 2B). No virus was found in the nasal swabs, saliva, milk, or any tissues of the 2 cows culled on days 6 and 12 postchallenge (Fig. 4A to C and E and Fig. S2B); however, from the cow culled on day 3 postchallenge, low levels of virus were detected in nasal swabs on days 2 and 3 postchallenge (Fig. 4A) and in its saliva on day 3 postchallenge (Fig. 4B), and viral shedding was detected in milk produced by the 2 virus-inoculated mammary glands that received 10^4^ and 10^6^ EID_50_ of virus, respectively. No virus was recovered in the milk collected from the gland that was challenged with 10^2^ EID_50_ of virus (Fig. 4C). The presence of virus was also detected in the trachea, in one lobe of the lung, and in the 2 high-dose virus-challenged mammary quarters of the cow culled on day 3 postchallenge (Fig. 4D), although the viral loads in nasal swabs, saliva, milk, and mammary glands of this cow were relatively lower than those from the corresponding samples from the control cow culled on the same day (Fig. 4A to D). Notably, the milk secreted by these 2 mammary glands had a milky appearance (Table 2), suggesting that the virus-caused damage to these glands may be relatively mild. Together, these results indicate that when the NT antibody titer in the serum of dairy cows is 16 or higher, the cows can still completely resist the attack of a high-dose H5N1 viral challenge delivered via intranasal and intramammary quarter inoculation, although the NT antibody in their milk is undetectable.

### Histological study of tissues collected from lactating dairy cows

Although evidence of viral replication was absent in cows challenged at 5 weeks post-first-vaccination and only limited replication was observed in the lungs and mammary glands from 1 of 3 cows challenged at 19 weeks p.v. (Fig. 3 and 4), it remains unclear whether viral challenge induced any pathological changes. To address this, we conducted histopathological examinations of the lungs and mammary glands collected from both unvaccinated and vaccinated cows euthanized on day 3 and/or 6 d postchallenge.

**Fig. 5.**
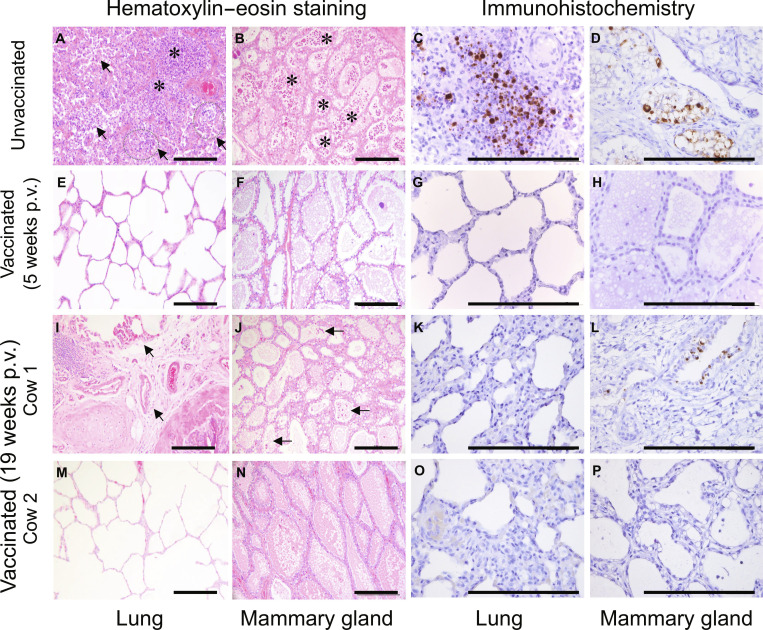
Histopathology of lungs and mammary glands of dairy cattle after challenge at different time points. Representative micrographs of H&E and IHC in the lungs and mammary glands of unvaccinated and vaccinated lactating dairy cows. H&E staining revealed accumulation of neutrophils, macrophages, and necrotic cellular debris (indicated by arrows) and marked necrosis and desquamation (indicated by asterisks) in the lung lobes and mammary quarters. (A to D) Control animal. (E to H) Vaccinated animals that were challenged at 5 weeks p.v. and euthanized on day 3 postchallenge. (I to L) Vaccinated cow challenged 19 weeks post-first-vaccination and euthanized on day 3 postchallenge. (M to P) Vaccinated cow challenged 19 weeks post-first-vaccination and euthanized on day 6 postchallenge. Scale bars, 100 μm.

In unvaccinated cows, histological examination of the lungs revealed extensive and severe obstruction of alveolar and bronchiolar lumens, characterized by the accumulation of neutrophils, macrophages, and necrotic cellular debris. Marked necrosis and desquamation of bronchiolar epithelial cells were also observed (Fig. 5A), indicative of severe acute bronchiolitis and alveolitis. In the mammary glands, widespread infiltration of neutrophils was observed in most of the alveolar lumens, accompanied by neutrophil degeneration and necrosis. Focal necrosis and sloughing of alveolar epithelial cells were also noted (Fig. 5B), suggesting acute suppurative mastitis with epithelial damage. Viral antigens were detected in both the virus-positive lungs and mammary glands of unvaccinated cows (Fig. 5C and D).

In vaccinated cows challenged at 5 weeks p.v., no major pathological changes were observed in either the lungs or mammary glands (Fig. 5E and F), and no viral antigens were detected (Fig. 5G and H). In vaccinated cows challenged at 19 weeks post-first-vaccination, those culled on day 3 postchallenge exhibited interstitial edema accompanied by mild inflammatory cell infiltration surrounding the bronchioles (Fig. 5I), suggesting a localized and nonsevere inflammatory response. In the mammary gland that had been directly inoculated with 10^6^ EID_50_ of virus, a few alveoli showed mild neutrophilic infiltration (Fig. 5J), indicating a localized or early-stage inflammatory response potentially associated with viral exposure. While no viral antigens were detected in the lungs (Fig. 5K), a few cells expressing viral antigen were detected in the mammary gland (Fig. 5L). In contrast, in the cow culled on day 6 postchallenge, in which no viral replication was detected (Fig. 4), no pathological changes were observed in the lungs or mammary quarters (Fig. 5M and N), and viral antigens were absent in all tested tissues (Fig. 5O and P).

### Rapid increase in NT antibodies in vaccinated cows after virus challenge

**Fig. 6.**
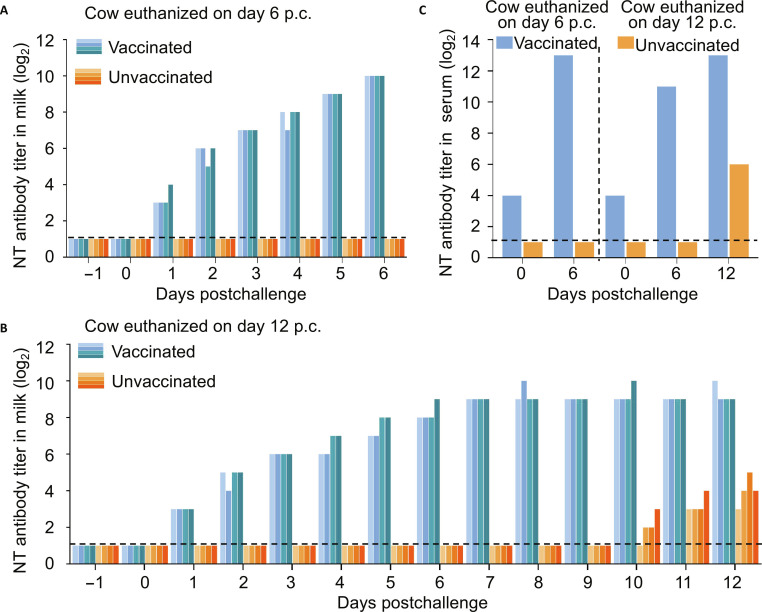
Neutralizing antibody responses in dairy cows at 19 weeks p.v. following viral challenge. Vaccinated and unvaccinated dairy cows were challenged with a dairy cow H5N1 virus via intranasal and intramammary routes at 19 weeks p.v. Neutralizing antibody (NT) titers in milk were determined from 2 cows euthanized on day 6 (A) and day 12 (B) postchallenge. Each bar represents the NT titer in an individual mammary gland. NT titers in serum (C) from both cows were also analyzed. The lower detection threshold is shown as a horizontal dashed line.

Of the 3 animals that were challenged at the 19th week after vaccination, 2 were completely protected. Notably, neutralizing antibodies were not detected in their milk, yet the high-dose intramammary inoculation of the virus still failed to replicate. We speculate that the vaccinated cows may have developed a strong immune response upon the virus stimulation. To test this hypothesis, we tested the NT antibodies in the milk that was collected from the vaccinated animals and control animals culled on days 6 and 12 postchallenge. We did not detect any NT antibody in milk samples obtained from the control cow culled on day 6 postchallenge (Fig. 6A), but we did detect the NT antibody on day 10 postchallenge in the milk collected from the control cow culled on day 12 postchallenge (Fig. 6B). In contrast, vaccinated cows exhibited a rapid and robust anamnestic response, with NT titers in their milk rising sharply on day 1 postchallenge and reaching 10 log_2_ on day 6 postchallenge (Fig. 6A and B). In the serum samples collected from the 2 vaccinated animals, NT antibody titers were greater than 10 log_2_ by day 6 postchallenge (Fig. 6C).

## Discussion

In this study, we developed an H5 mRNA–LNP vaccine and evaluated its safety, immunogenicity, and protective efficacy in lactating dairy cows. We demonstrated that the mRNA vaccine is safe for use in cattle, as no adverse clinical signs were observed and the milk yield was unaffected following both the prime and booster vaccinations. Importantly, the vaccine was highly immunogenic in dairy cattle, inducing a robust antibody response after 2 doses of vaccine. Cows immunized with the vaccine were fully protected against high-dose H5N1 viral challenge when the antibody titers in serum and milk were at their peak. Remarkably, 19 weeks post-first-vaccination, when the serum antibody level dropped to a very low level and the NT antibody level was undetectable in milk, 2 of the 3 cows were fully protected even when they were simultaneously challenged with high doses of the H5N1 virus via their nasal and mammary glands. This study not only demonstrates that the HA-based H5 mRNA–LNP vaccine effectively protects dairy cattle against H5N1 virus infection but also provides important insights into the practical application of this vaccination strategy in lactating dairy cattle.

Studies have shown that the COVID-19 mRNA vaccine induces both humoral immunity and cellular immunity [34]. Souza et al. [31] observed that 500-μg H5 mRNA–LNP inoculation triggered a significantly higher level of proliferating CD8^+^ T cells in calves compared to unvaccinated ones, and we believe that mRNA vaccine also elicited a T cell response in lactating dairy cattle, although we did not evaluate cellular immunity in our study. Our antibody duration analysis indicated that NT antibodies in vaccinated cows’ milk became undetectable by the 15th week postimmunization. When the animals were challenged at the 19th week postimmunization, the control group animals only exhibited detectable NT antibodies in their milk on day 10 postchallenge, but in the vaccinated animals, the NT antibodies were first detected on day 1 postchallenge, rapidly peaking on day 7 postchallenge, and virus replication was totally inhibited. Although the cellular immune responses in these animals could not be assessed because of the unavailability of species-specific reagents, we hypothesize that the rapid antibody production and efficient viral clearance observed in vaccinated animals are likely attributable to the memory response of B cells.

The antibody titer in serum has long been used as an important parameter for monitoring the immune status of animals and humans after influenza vaccine inoculation. In humans, an HI titer of 40 induced by inactivated vaccine is generally considered to confer approximately 50% protection against seasonal influenza virus infection [35]. In poultry, immunization with inactivated vaccines confers complete protection when the HI antibody titer against H5 subtype challenge viruses reaches or exceeds 16 [36–38]. For H5 mRNA vaccine, the protective threshold varies across species. Chiba et al. [23] reported that mice were fully protected against challenge with the dairy-cow-derived H5N1 virus when serum NT titers ranged from 20 to 40 after an mRNA vaccine inoculation, with no virus detected in the vaccinated mice; Hatta et al. [28] reported that even a high NT titer of 1,280 in serum did not confer 100% protection against H5N1 virus infection. In the present study, the 2 cattle that were challenged at 19 weeks p.v. and were completely protected had HI antibody titers of only 8 and 16, respectively, and an NT antibody titer of 16 in their sera. In China, when the HI antibody titer against highly pathogenic influenza virus in vaccinated poultry declines to 16, the birds must be revaccinated [37–39]. Current research indicates that if this vaccination strategy were to be applied to H5N1 influenza prevention and control in cattle, HI antibody and NT antibody titers of 16 could serve as appropriate parameters for determining the need for revaccination of dairy cattle.

In summary, our findings provide strong evidence that the mRNA vaccine is safe, able to trigger robust immune responses, and offers complete protection against H5N1 virus challenge in lactating dairy cows. In addition to H5-inactivated and DNA vaccines, the HA-gene-based mRNA vaccine could provide another option for rapid and effective control of emerging H5N1 viruses in dairy cattle.

## Materials and Methods

### Facility and ethics statements

The procedures involving live HPAI viruses were performed within certified BSL-3 and ABSL-3+ laboratories at the Harbin Veterinary Research Institute (HVRI), Chinese Academy of Agricultural Sciences (CAAS). These facilities are authorized by the Ministry of Agriculture and the China National Accreditation Service for Conformity Assessment for work with HPAI viruses. All animal experiments involving dairy cattle were approved by the Institutional Animal Care and Use Committee of HVRI, CAAS, under protocol number 240531-01-GJ.

### Viruses

The challenge dairy cow H5N1 virus (DC/24), the first strain isolated from a dairy cow in Texas, USA, was generated as previously described [2]. Vaccine strain H5-Re14, with surface gene segments from A/whooper swan/Shanxi/4-1/2020 (H5N8) and its 6 internal gene segments from A/Puerto Rico/8/1934 (H1N1, PR8) were maintained in our laboratory. Dairy cow H5N1 vaccine strain, with HA and NA genes from DC/24 and its 6 internal genes from PR8, was rescued as previously described [27]. All viruses were cultured in specific-pathogen-free embryonated chicken eggs. The infectious dose (EID_50_) was measured in eggs and was determined using the method of Reed and Muench.

### Cells

MDCK (Madin–Darby canine kidney) and human embryonic kidney 293T cells were obtained from American Type Culture Collection (https://www.atcc.org/). MDCK cells were propagated in DMEM (Dulbecco’s modified Eagle’s medium) containing 6% newborn calf serum, and 293T cells were maintained in DMEM supplemented with 10% fetal bovine serum. All cell lines were grown at 37 °C with 5% CO_2_.

### mRNA–LNP vaccine production

A cattle-codon-optimized HA gene, encoding a protein identical to that of the clade 2.3.4.4b H5N8 virus A/whooper swan/Shanxi/4-1/2020, the donor of the vaccine strain H5-Re14, and the vaccine strain used for control of H5 viruses in China [36], was cloned into the pXT7 plasmid to generate pXT7-H5. Following large-scale plasmid preparation, pXT7-H5 was linearized with Xba I (New England Biolabs, USA) and purified using the MiniBEST Agarose Gel DNA Extraction Kit Ver.4.0 (TaKaRa, China). Transcription was performed in vitro using the HiScribe T7 ARCA mRNA Kit (with tailing) (New England Biolabs, USA) according to the manufacturer’s protocol, with the linearized plasmid as the template. Modified nucleotides pseudo-uridine-triphosphate and 5-methyl-cytidine-triphosphate (APExBIO, USA) were incorporated during transcription, and a Cap0 structure was added to the 5′ end of the transcript. Residual plasmid DNA was removed by deoxyribonuclease I digestion, followed by polyadenylation to add an approximately 150-nucleotide polyadenylate tail. The resulting mRNA was purified using the Monarch RNA Cleanup Kit (50 μg) (New England Biolabs, USA) and stored at −80 °C until use. Purified mRNA was encapsulated into LNPs using a formulation adapted from previously reported methods [24,40,41]. The lipid mixture, consisting of 1,2-distearoyl-*sn*-glycero-3-phosphocholine, cholesterol, mPEG-DMG-2K lipid, and ionizable lipid in ethanol at a molar ratio of 17:80:3:100, was mixed with 50 mM citrate buffer (pH 4.0) containing mRNA (150 ng/μl) at a 1:3 (v/v) ratio using a microfluidic mixer (INano E, Micro&Nano Technology Inc., China). The resulting mRNA–LNPs were diluted with sterile Ca^2+^/Mg^2+^-free phosphate-buffered saline (PBS), subsequently concentrated by centrifugal filters (30-kDa molecular weight cutoff; Millipore, USA), and sterilized through a filter (0.22 μm). The final preparation was adjusted to the desired mRNA concentration in PBS and stored at 4 °C until use.

### Protective efficacy evaluation in dairy cattle

Chiba et al. [23] reported that a 10-μg dose of the mRNA vaccine conferred complete protection against H5N1 virus infection in mice, and on the basis of this finding and the practical, species-to-species dose conversion methodology described by Nair et al. [42], the estimated effective dose for cattle is projected to be in the range of 500 to 1,000 μg. Therefore, in this study, we used 500 μg of mRNA to vaccinate dairy cattle.

Lactating Holstein cows, 3 to 5 years of age, obtained from a local dairy farm, were used in the challenge experiment. Six cows were intramuscularly immunized with two 500-μg injections of the mRNA vaccine spaced 3 weeks apart. The milk yield was recorded daily beginning 3 d before vaccination, and milk and blood were collected weekly to assess the immunogenicity of the vaccine. For the challenge study, 3 vaccinated cows were challenged at 5 weeks p.v., and the remaining 3 were challenged at 19 weeks p.v., along with 3 age-matched, unvaccinated lactating cows for each group. Prior to viral challenge, all animals were housed individually in ABSL-3+ facilities for a 2-d acclimation period. Each cow was kept in a separate stall measuring 1.8 m × 2.5 m. For viral challenge, the cows were anesthetized with xylazine (0.2 mg/kg, intramuscular injection) and inoculated via both intranasal and intramammary routes. A 5-ml syringe fitted with a straight oral gavage needle (16 gauge × 110 mm) was used for virus delivery. For intranasal inoculation, each cow received 2 × 10^6^ EID_50_ of H5N1 virus in 2 ml of inoculum, with 1 ml administered into each nostril. For intramammary inoculation, after teat disinfection, 3 quarters (left rear, right front, and right rear) were inoculated with 10^2^, 10^4^, and 10^6^ EID_50_ of virus, respectively, with 1 ml administered into each quarter via teat. Nasal swabs, saliva, and milk from each mammary gland were collected daily starting on day 1 postchallenge to assess the presence of virus. On days 3, 6, and 12 postchallenge, one cow from each group was humanely euthanized. Tissue samples for viral titration were obtained from the upper respiratory tract (nasal turbinates, soft palate, tongue root, larynx, and tonsils), submandibular lymph nodes, salivary glands (sublingual, submandibular, and parotid), trachea, bronchi, all 6 lung lobes, and the 4 mammary quarters. Rectal body temperatures were recorded daily throughout the experiment.

### Antibody isotype determination

Antibody isotypes induced by the mRNA vaccine in bovine serum and milk were determined 2 weeks after the second immunization using the enzyme-linked immunosorbent assay (ELISA) method (indirect ELISA). Samples from unvaccinated dairy cattle served as negative controls. The procedure was as previously described [2]. Briefly, purified H5-Re14 vaccine virus was applied to coat plates (200 μl per well), followed by incubation at 4 °C for 10 h. After coating, plates underwent 4 washes with PBS–0.05% Tween 20 (PBST) and were then incubated in PBST with 5% skim milk for 1.5 h at 37 °C to block nonspecific binding. Serial 4-fold dilutions of serum and milk samples were added to the wells (200 μl per well) and incubated for 1.5 h at 37 °C. After washing the plates 3 times with PBST, sheep antibovine IgG or IgA antibodies (Bio-Rad, USA) were added as secondary antibodies and incubated for 1 h at 37 °C. This was followed by incubation with horseradish-peroxidase-conjugated anti-sheep IgG antibody (Sigma-Aldrich, USA) under the same conditions. Color development was achieved using 3,3′,5,5′-tetramethylbenzidine substrate (Thermo Fisher Scientific, USA), and 2 M H_2_SO_4_ was added to stop the reaction. Absorbance was then recorded using a microplate spectrophotometer at 450 nm.

### Antibody analysis

Antibody titers in serum were evaluated by using HI and NT assays, whereas antibody levels in milk were assessed exclusively using the NT assay. Since the antigenicity of the H5-Re14 vaccine strain is identical to that of the challenge virus [2], all assays were conducted using the H5-Re14 virus. For the HI assay, one volume of bovine serum was first absorbed with 2 volumes of 50% chicken red blood cells (cRBCs) at 22 to 24 °C, followed by centrifugation of the mixture and collection of the supernatant. Then, 25 μl of serum was serially diluted 2-fold across the rows of a cell plate, after which 25 μl of PBS containing 4 hemagglutinating units of virus was dispensed into the bottom of each well. After incubating at 22 to 24 °C for 30 min, 1% cRBCs (25 μl) was added. After incubating the samples for another 30 min, the titer was taken, as the highest dilution showing full inhibition of cRBC agglutination. For the NT assay, serum and milk samples were first centrifuged at 1,200 rpm for 2 min and then heat-inactivated at 65 °C for 1 h to eliminate residual live virus [43]*.* Two-fold serial dilutions of each sample (50 μl per well) were dispensed across the wells of a plate. Then, each well was added with 50 μl of PBS containing 100 EID_50_ of H5N1 virus. Subsequently, the plate was incubated was carried out at 37 °C for 1 h to enable the virus to interact with the antibodies. Following incubation, the mixtures were transferred onto a new plate preseeded with MDCK cells. After a further incubation, the plate were washed 4 times and refilled with minimum essential medium with TPCK (*N*-tosyl-L-phenylalanyl chloromethyl ketone)-treated trypsin (0.5 μg/ml; Sigma-Aldrich, USA). To assess viral replication, a hemagglutination experiment was conducted at the end of the experiment. Neutralizing titer is the highest serum dilution at which viral replication is prevented.

### Histological study

Lung and mammary quarter tissues from infected dairy cows were collected and fixed in 4% formalin for 2 to 3 d [2]. Following fixation, the samples were underwent paraffin embedding, and 4-μm-thick tissue sections were sliced using a microtome. These sections were then subjected to hematoxylin and eosin (H&E) staining and immunohistochemistry (IHC) analysis. For IHC analysis, a laboratory-made monoclonal antibody against the influenza nucleoprotein, derived from mouse, was used as the primary antibody. Final detection was performed using a secondary antibody (Thermo Fisher Scientific, catalog no. 32230).

## Data Availability

All data are available in the main text or the Supplementary Materials.
