# Supplementary material for: Protective Efficacy of a Hemagglutinin-Based mRNA Vaccine Against H5N1 Influenza Virus Challenge in Lactating Dairy Cows
Source: Research (Wash D C). 2026 Jan 26;9:1104. doi: 10.34133/research.1104 (PMC12833822; doi:10.34133/research.1104)
Supplement: Supplementary 1 — Figs. S1 and S2 [file research.1104.f1.pdf]

Fig. S1

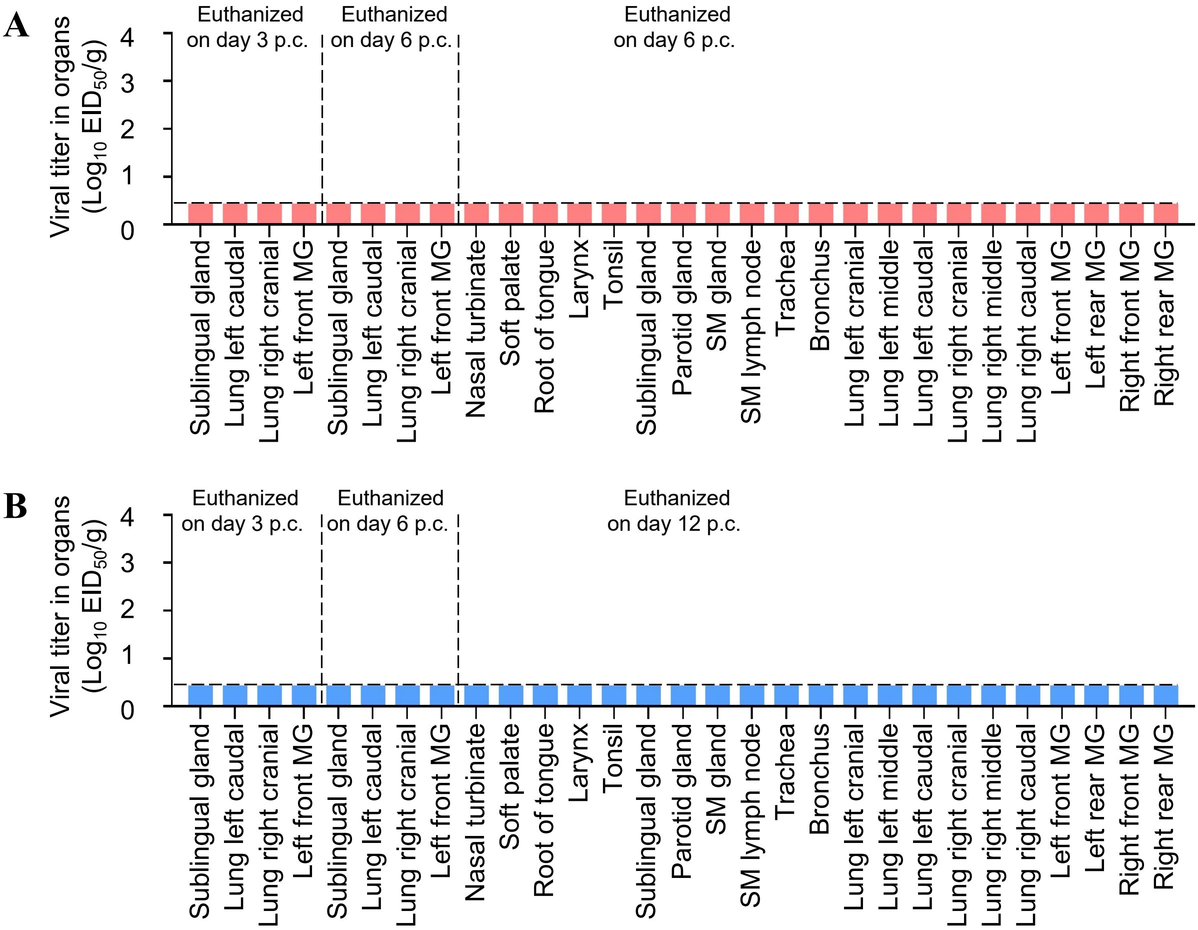

Fig. S1. Protective efficacy of the H5 mRNA–LNP vaccine in lactating dairy cows 5 weeks post-vaccination. Vaccinated and unvaccinated dairy cows were challenged with a dairy cow H5N1 virus via intranasal and intramammary routes. A dose of 2 × 10^6 EID_{50} of virus was administered intranasally (1 mL per nostril), and different doses were administered to individual mammary quarters via the teat: 1 mL of PBS into the left front (LF) quarter, 1 mL (10^2 EID_{50}) into the left rear (LR) quarter, 1 mL (10^4 EID_{50}) into the right front (RL) quarter, and 1 mL (10^6 EID_{50}) into the right rear (RR) quarter. Tissues were collected at the indicated days and titrated in embryonated chicken eggs. (A) Viral titers in different tissues of unvaccinated cows euthanized on day 3, 6, and 12 post-challenge (p.c.). (B) Viral titers in different tissues of vaccinated cows euthanized on day 3, 6, and 12 p.c. SM, submandibular, MG, mammary gland.

Fig. S2

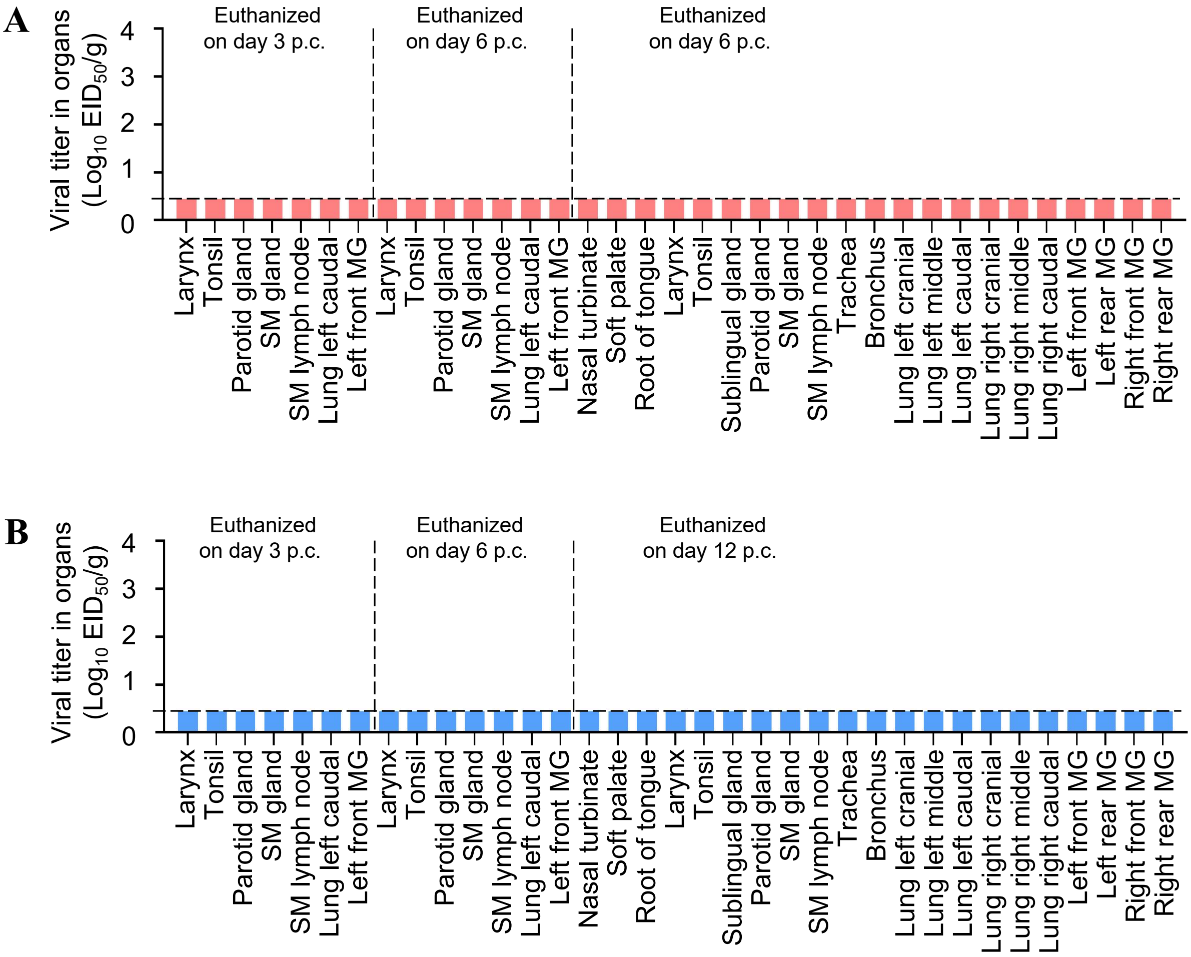

Fig. S2. Protective efficacy of the H5 mRNA–LNP vaccine in lactating dairy cows 19 weeks post-vaccination. Vaccinated and unvaccinated dairy cows were challenged with a dairy cow H5N1 virus via intranasal and intramammary routes. A dose of 2×10^6 EID_{50} of virus was administered intranasally (1 mL per nostril), and different doses were administered to individual mammary quarters via the teat: 1 mL of PBS into the left front (LF) quarter, 1 mL (10^2 EID_{50}) into the left rear (LR) quarter, 1 mL (10^4 EID_{50}) into the right front (RL) quarter, and 1 mL (10^6 EID_{50}) into the right rear (RR) quarter. Tissues were collected at the indicated days and titrated in embryonated chicken eggs. (A) Viral titers in different tissues of unvaccinated cows euthanized on day 3, 6, and 12 post-challenge (p.c.).. (B) Viral titers in different tissues of vaccinated cows euthanized on day 3, 6, and 12 p.c. SM, submandibular, MG, mammary gland.
